# Status of Clinical Neuropsychology Training in Finland

**DOI:** 10.3389/fpsyg.2022.860635

**Published:** 2022-03-03

**Authors:** Laura Hokkanen, Hanna Jokinen, Kati Rantanen, Taina Nybo, Erja Poutiainen

**Affiliations:** ^1^Department of Psychology and Logopedics, Faculty of Medicine, University of Helsinki, Helsinki, Finland; ^2^Division of Neuropsychology, Neurocenter, Helsinki University Hospital and University of Helsinki, Helsinki, Finland; ^3^Department of Rehabilitation and Psychosocial Support, Tampere University Hospital, Tampere, Finland; ^4^Rehabilitation Foundation, Helsinki, Finland

**Keywords:** clinical neuropsychology, Finland, specialization training program, history, university

## Abstract

This paper provides information on different training models within clinical neuropsychology in Finland. Systematic specialization training program began in Finland in 1983. It was first organized mainly by the Finnish Neuropsychological Society and since 1997 by the Finnish universities. At present, close to 400 clinical neuropsychologists have completed the training. The number of professionals still does not cover the needs of the country (population 5.5 million, area 338,440 km^2^), and geographical disparities are a constant concern. The training models in Finland have changed over the years and will continue to evolve. Specialization training can be organized by national societies or by universities. It can lead to an academic degree or a diploma. It can be linked to doctoral studies or form a parallel track. Financial model can involve student fees or be governed by ministries (such as the Ministry of Education or Ministry of Health). This paper describes and compares different strategies in education that have impact on the output of professionals. One model does not fit all, or even one country at all times. The strategies of the stakeholder ministries can change over time. The experiences from Finland can be useful for other countries that are developing their models. The estimated need of practitioners and the educational resources including the available financial models for training differ between countries. The guiding principles in specialist training should focus on the advanced competencies expected from the neuropsychologist when entering the profession.

## Introduction

Neuropsychology in Finland has its roots in European research and practice, especially the nineteenth century German tradition of experimental psychology and psychophysics (Hokkanen et al., 2016). Clinical neuropsychology developed as its own area of practice during and after the II World War in Finland, as in several other countries in Europe (Hessen et al., 2018a; Hokkanen et al., 2020). The first neuropsychologists, although the word did not exist at the time, were psychologists who served in the rehabilitation of war veterans, and gradually the practice expanded to civilian health care (Laaksonen, 1987). In Finland as in the other Nordic countries today, neuropsychologists work in hospital settings, outpatient clinics and rehabilitation institutes, educational settings, and in private practice (Norup et al., 2017). The areas of work have expanded from the neurological disorders (e.g., stroke, traumatic brain injury, dementia, epilepsy) to developmental and neuropsychiatric conditions (e.g., attention deficit hyperactivity disorder, dyslexia, autism) covering the full lifespan (Hokkanen et al., 2016; Norup et al., 2017; Turunen et al., 2019).

Education and training of clinical neuropsychologists is heterogeneous ranging worldwide from master’s programs to post-doctoral training (Grote and Novitski, 2016; Hokkanen et al., 2019). One third of the countries in Europe have no model in place for specializing in clinical neuropsychology (Hokkanen et al., 2019). Countries where specialist training models are currently only emerging include some with a small population and language base such as Estonia (Randver et al., 2015), but also some with a large population and a long history in neuropsychology such as France (Branco Lopes et al., 2021). In Scandinavia, only 42% of psychologists working in neuropsychology report to have been approved as specialists (Norup et al., 2017). There are bottlenecks in educational availability and since the title of clinical neuropsychologist is not protected by law in any of the Nordic countries (Kasten et al., 2021), also psychologist with less training enter the field. An established training can serve in strengthening the profession and its independent status (Hokkanen et al., 2019; Kasten et al., 2021). There is still a considerable need to develop training models in Europe to ensure consistent professional competencies and quality of neuropsychological practices.

This paper describes the models that have been in use in clinical neuropsychology training in Finland since early 1980s. The models and strategies have changed over the years, and the pros and cons of each are discussed.

## Training of a Clinical Neuropsychologist in Finland

### Education and Training to Become a Licensed Psychologist

Before applying to specializing training in neuropsychology, one has to be licensed as a psychologist. For licensing, a master’s degree in psychology is required. In Finland, as elsewhere in Europe where the so-called Bologna process^1^ and EuroPsy model has been adopted, the academic studies are measured using the European Credit Transfer and Accumulation System (ECTS), where one academic year is defined as 60 ECTS-credits, corresponding to roughly 15:00–18:00 h of study; 1 ECTS means 27 h of study (Lunt et al., 2015). To graduate from psychology, a bachelor’s degree (180 ECTS, typically 3 years) followed by master’s degree (150 ECTS, typically 2.5 years) are required. In Finland, master level studies offer courses of professional skills on several areas of psychology - clinical, developmental and neuropsychology–and there is no specialization within the master’s degree. Specific to the study program in Finland is a 5-month full-time compulsory practical training period, an internship.

After completing the master’s degree, the students apply for a license to practice as a psychologist from The Finnish National Authority for Welfare and Health. The license is granted directly after completion of the degree. Under Finnish law, licensing is required for health care professions, including psychology, and the practice of these professions is restricted to licensed professionals only.

Finland has adopted the European professional qualification standards established by the European Federation of Psychologists’ Associations (EFPA). The bachelor’s and master’s study program follows the guidelines in content and depth (Lunt et al., 2015). The so-called EuroPsy diploma^2^ requires a 1 year supervised practical training period, so students graduating from Finland can apply for the diploma after few additional months of clinical work.

### Specialist Education and Training in Clinical Neuropsychology

#### Post-licensing Continuous Education in Clinical Neuropsychology

Training of professionals is based on continuing education (CE). The Finnish Neuropsychological Society has organized seminars and courses for psychologists in Finland ever since its foundation in 1979. The objective of the courses was to increase clinical skills in neuropsychological assessment, methodology, and interventions. Right from the start neuropsychologists in Finland have been an important provider of rehabilitation services and in 1982, in collaboration with the Social Insurance Institution of Finland (Kela) which reimburses rehabilitation, the Finnish Neuropsychological Society started to keep a record of certified psychologists qualified to carry out neuropsychological rehabilitation subsidized by Kela (Nybo et al., 2011; Hokkanen et al., 2016). This work was later continued by a specific Certification Board. The certification required a certain number of seminar hours, supervision hours and work experience (see Table 1). While the courses had a wider scope, the certification focused on rehabilitation and was not required for neuropsychological assessments.

**TABLE 1:** Post-master specialization in Finland.

	CE model	Society program	Degree program	Diploma program
Available	1983–2007	1983–1997	1997–2015	2016–>
Organizer	Variable	The Finnish Neuropsychological Society	University	University
Total length	Undetermined	4 years	4 years (target)	3 years
Required course work in neuropsychology and related sciences	240 h	47 ECTS	55 ECTS	55 ECTS
Required research training	None	None	Research methods 15 ECTS Licentiate thesis 40 ECTS	Research methods 5 ECTS Final thesis 10 ECTS
Required work experience	3 years full time in clinical neuropsychology	4 years full time in clinical neuropsychology with supervision	4 years full time in psychology, of which 3 years in clinical neuropsychology with supervision^b^	4 years full time in psychology, of which 3 years in clinical neuropsychology with supervision^c^
Required supervision^a^	60 h	90 individual or 120 group hours	90 individual or 120 group hours	90 individual or 120 group hours
Funding model	Participant fees	Student fees	Ministry of Education (fully)	Ministry of Education (partly) + student fees
Pros	- Flexible for the students - Flexible distribution of resources for organizing courses	- Deep understanding of the clinical context in professionals planning the training - High motivation in professionals planning the training - Clear curriculum: the decision to accept or reject the selection of courses known beforehand	- University is an acknowledged provider of higher education - Infrastructure available - Degree studies free for students in Finland - Studies easily extendable to a doctorate	- University is an acknowledged provider of higher education - Infrastructure available - Literature review useful and feasible for the students - Strict time limit for studies provides incentive to complete the program
Cons	- If used for certification, requires resources for confirming the acquired competencies - Low control over the covered competency areas - Low control over the acquired skill level - The decision to accept or reject the selection of courses came from the Certification Board afterward	- No supporting infrastructure to organize education - Very time consuming for a small society board - Other society activities suppressed - No official status within the educational system	- No available post-master degree in the Bologna model except doctorate - Financial model depended on funding from the Ministry of Education, which was later discontinued (as the degree was not recognized by the Bologna model) - Low incentive for the students to complete their studies in target time - Required empirical research challenging for some students to complete	- Students pay a fee for specialist expertise vital for health care - Financial model creates difficulties in long-term planning of the program - Limited intake results in delayed admission for some applicants

*Comparison of different training models over the years. European Credit Transfer and Accumulation System (ECTS) has been in use since 2005. Credits prior to that have been translated into ECTS for comparison. One academic year (full time study) equals 60 ECTS.*

*CE, continuing education.*

*^a^Only supervision given by accredited neuropsychologists accepted. Supervision can be received either individually or in small groups. Group supervision corresponds 0.75× individual supervision hours.*

*^b^Counted as 10 ECTS in the program*

*^c^not counted as credits in the program.*

Continuing education-courses however are not recommended as the way to specialize (Bornstein, 1988) and as the curriculum-based program was developed (see Neuropsychological Society as the Organizer of Post-Master Specialist Training Program 1983–1997), the CE-based system was discontinued in 2007. After that only neuropsychologists who have completed the full program were certified for subsidized rehabilitation services (Finnish Psychological Association, 2006).

Continuing education-training in neuropsychology still continues and has an important role in health care. Many organizations and institutions offer courses that are useful for clinical psychologists who need additional information on neuropsychology, and they are very popular and in high demand. CE-courses do not qualify for the certification for neuropsychological rehabilitation or give the specialist diploma.

#### Neuropsychological Society as the Organizer of Post-master Specialist Training Program 1983–1997

The first curriculum-based program in clinical neuropsychology started as the activity of the Finnish Neuropsychological Society. Programs were approximately 4 years of length, 1983–1987, 1988–1992, and 1992–1995 (Nybo et al., 2011). The courses were planned and organized by the Finnish Neuropsychological Society and to ensure the scientific level of the training, a steering board was established that included members from two separate universities in Finland. The training was nation-wide, and students came from different parts of the country. Courses were organized as full-day seminars and the students paid a fee for participation. The students were working as clinical practitioners at the same time and work was linked with professional supervision. In all, 133 psychologists completed one of the three early programs and became certified in clinical neuropsychology, 78 of them working mainly with adults, 55 with children (Hokkanen et al., 2016).

#### University as the Organizer of Post-master Specialist Degree Program 1997–2015

In 1997, curriculum-based specialization programs were launched in Finland in five different areas of psychology practice: health psychology, developmental psychology, psychotherapy, work- and organizational psychology, and neuropsychology (Järvinen, 2008, p. 31). Programs were fully organized by the universities and all led to the licentiate degree (an interim degree between master’s and doctorate) of 120 ECTS. All programs were nation-wide, and coordinated by Psykonet, a national network of all six departments of psychology in different universities across Finland.^3^ In this model, students were able to enroll to any one of the Finnish universities to study in their chosen program regardless of where the teachers were employed. They chose their “home university” based on where they lived and the group they wanted to join to complete the research required. The network was ahead of its time organizing remote video- and web-based teaching already since 1997 (Järvinen, 2008, p. 47). Like all university degree studies in Finland, tuition was free for students and there was no strict time limit in the graduation although 4 years was the target time.

Licentiate thesis in the program (40 ECTS) consisted of one publishable article reporting an empirical study in clinical neuropsychology. Many papers were published in scientific peer reviewed journals, international or national. All thesis were evaluated, usually by two independent reviewers, before approval by the faculty as part of the degree. The work preceding publication was carried out in research groups with variable resources. Some were large active groups in university departments, while some were small *ad-hoc* projects arising from clinically relevant questions at the workplace of the student (Järvinen, 2008, p. 54). Courses on research methods (ethics, statistics, imaging methodology, research paradigms, etc. 15 ECTS required) necessary to complete the study were offered by different universities and coordinated by Psykonet.

The close link between theoretical study and clinical work was emphasized and achieved as the program was part-time. All students simultaneously worked as practitioners. The work experience, as well as supervision, was a requirement for the qualification and degree (Table 1). In all, 237 psychologists in Finland have completed a licentiate degree in clinical neuropsychology since 1995.

#### University as the Organizer of Post-master Specialist Diploma Program 2016 Onward

As the legislation related to specialist education in Finland changed in 2015 and the licentiate degree ceased to be supported by the ministry funding, the training model for clinical neuropsychology was modified. The degree status changed into a diploma status and the students were again required to pay a fee for their tuition.

The program is currently organized by the faculty of Medicine at the University of Helsinki in collaboration with the Psykonet network. Students still come from all over Finland, but they enroll to the University of Helsinki. Other universities in Finland have similar responsibility of other specialization fields. In its current form the program (70 ECTS in total) is a 3-year program where students gain a time-limited access to the studies. The content is described in more detail by Hokkanen et al. (2016) and the program website https://www.helsinki.fi/en/faculty-medicine/education-and-studying/postgraduate-professional-education/specialisation-programme-neuropsychology.

Eight to ten 2-days seminars are organized each academic year (28 in total) but not all seminars are compulsory to all. The students can select courses based on their interests and personal emphasis on adult or on child neuropsychology, although a certain core content over the entire lifespan is obligatory. In addition to the seminars, there are regular groups for clinical work supervision and thesis supervision. The required literature is covered in various ways; in addition to book exams, students write essays and reviews based on recent relevant articles. Each student also prepares and presents a lecture on a topic of their choosing to other students and keeps a portfolio on professional development.

**FIGURE 1:**
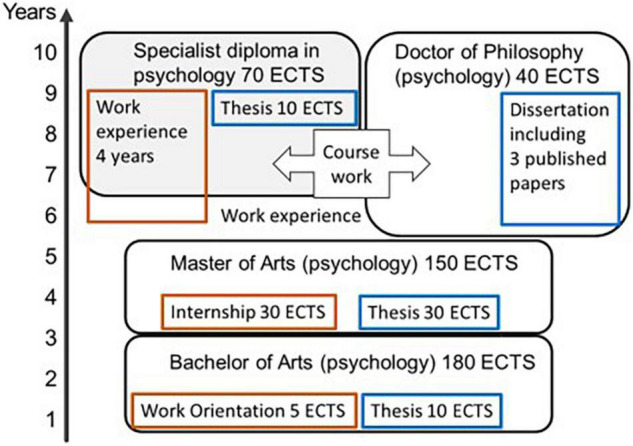
The track for specialist education and the track for doctorate in Finland currently. Both tracks build upon a master’s degree and part of the course credits from one can be counted toward the other. Clinical work practice requirements on different levels in brown, scientific writing requirements in blue. ECTS, European credit transfer and accumulation system, 60 ECTS equal one full-time academic year.

Instead of the licentiate thesis, the students write a final thesis of a smaller scale (10 ECTS). It involves a systematic literature review on a question relevant to clinical practice or an empirical research paper. Research papers are preferred by students who simultaneously pursue doctoral studies and they are published in international peer-reviewed journals. The reviews are mostly published in a non-peer reviewed open access journal by the University of Helsinki, Neuropsy Open (journals.helsinki.fi/neuropsyopen). The accompanying methodological studies (five ECTS) focus on skills in online literature search, systematic review practices, and critical appraisal of research quality. The specialist track in the current model does not expand to doctoral studies as easily as the previous degree model did, but content can be selectively shared between tracks (see Figure 1).

Admission to the program is every 3 years. In 2016, 41 psychologists (out of 100 applicants) were admitted and additionally some transferred from the earlier degree program to the diploma program. In all, 52 psychologists have currently completed the diploma program. In 2019, 53 psychologists (out of 141 applicants) were admitted to the program and they will complete in 2022. All curriculum-based models combined the current number of trained clinical neuropsychologists is 373 (Finnish Neuropsychological Society, 2021). Most employers, especially in the public sector, recognize the specialist qualification and require the training from neuropsychologists regardless of if the work involves assessment or rehabilitation. Table 1 describes all the models and their perceived pros and cons.

### Post-specialization Education and Training

In Finland it is mandatory by law for everyone working in the health care sector to maintain their high level of expertise, and employers are required by law to allow adequate time for this (recommendation of at least 3–10 days of continuing education each year). Practitioners in general are very active in personal and professional development, and especially those working in clinical neuropsychology acknowledge the rapid turnout of new information in neurosciences and the need for continuous update of education. Hospital districts offer local training to their employees, and many participate in international conferences by e.g., International Neuropsychological Society, where CE-workshops and special lectures are available.

Continuing personal development (CPD) for all practicing psychologists is recommended in the EuroPsy model (Lunt et al., 2015). In Finland, there is currently no official requirement for this by national authorities. To renew the EuroPsy diploma (it is valid for 7 years) the psychologist must present CPD documentation to the National Awarding Committee, appointed by EFPA.

For those wishing to serve as supervisors in clinical neuropsychology, special requirements apply and the supervisor certificate is awarded by the Certification Board (Hokkanen et al., 2016). In addition to the specialist training program in clinical neuropsychology and accumulation of added supervised clinical practice, the supervisor is recommended to obtain further training in supervisory skills. A supervisor training program has been offered by the Finnish Neuropsychological Society and is currently 12 ECTS. There are also general (without the emphasis on clinical neuropsychology) supervisor training courses offered by other institutions in Finland.

## Discussion

Specialist education and training in clinical neuropsychology can be organized in many ways. Finland has experience in four different models: CE-model, a program organized by a national neuropsychological society, a degree program organized by universities, and finally a diploma program organized by universities. All have had their pros and cons. When considering these models and trying to select the best for a particular country without an existing model, some points especially are worth discussing.

### Practitioner Supply and Demand

One point to consider is the required efficiency of training, that is, the number or specialists needed to graduate per year. This should be compared to the estimated need of practitioners. In Europe, the numbers of clinical neuropsychologists have been reported to vary between 1 per 10,455 (Denmark) and 1 per 704,000 (Serbia) (Hokkanen et al., 2019). Figures reported from countries with a long and well-developed tradition in clinical neuropsychology include 1 per 19,444 in Canada, 1 per 40,885 in Australia, and 1 per 80,250 in United States (Grote and Novitski, 2016). In Scandinavian countries that have similar history in the development of professional clinical neuropsychology (Hessen et al., 2018a), the estimated numbers of trained neuropsychologists have reported to be 1 per between 10,000 and 20,000 (Hokkanen et al., 2019).

The desired number of practitioners will depend on the size of the population and the geographical area to be covered. It will also depend on the health care system and clinical practices. Preferably, the number of neuropsychologists should be in line with the number of other relevant professionals such as neurologist or psychiatrist [see Kasten et al. (2021) for comparison]. Low number of trained specialists can partly, or temporarily, be compensated by organizing the work accordingly. By focusing on a consultative role, instead of direct client work, one neuropsychologist can guide the work of many professionals [see Ghag et al. (2021)] thus reaching more patients or clients by proxy. In Finland, neuropsychological supervision of other professionals seems to be a less frequent activity compared to other Nordic countries (Norup et al., 2017) and the neuropsychologists are directly involved in the assessment as well as rehabilitation of clients. This increases the need of trained specialists. In the United States, neuropsychological testing and scoring are often carried out by assistants, while the trained neuropsychologist is responsible for the interpretation of the results and communication of the findings (Bornstein, 1988; Axelrod et al., 2000). While it may save time, this model is not widely in use or recommended in Europe. Ideally, the number of trained specialists in time rises to meet the true need.

In Finland, the current density of clinical neuropsychologists (the number of trained specialists compared to the population of 5.5 million) is 1 per 14,745. While at the high end in global comparison, this amount of specialist has been considered inadequate (Finnish Psychological Association, 2016; Ministry of Education and Culture, 2019; Finnish Psychological Association, 2021) and especially because of the large geographical area (338,440 km^2^) disparities between different parts of the country are a concern. There is therefore a constant pressure to increase the intake and/or output of students. Short training times produce specialists quicker, but the quality of training is paramount. Enough time should be allowed for the development of the competencies expected from the graduating neuropsychologists as they enter the profession. Admitting more students in the program also produces specialists quicker, but the pedagogical quality should not be compromised. Higher intake requires more resources for teaching and supervision. The EuroPsy specialist certificate, currently available in work and organizational psychology and psychotherapy, require a 3-year post-master training with at least 90 ECTS of advanced study^4^ which suggests that acquisition of advanced skills requires time. Clinical neuropsychology tends to be a popular field among psychologists, so lack of motivated applicants is not a problem.

### Financial Models

The costs of organizing a training program come from many sources. Paying the salaries of individual lecturers and renting the premises is a beginning. In universities, cost models are calculated to include variable size overheads e.g., for the use of offices and auditoriums, information technology, general administration, and secretarial assistance. Salaries for professors and university lecturers include time to plan the full curriculum, course syllabus, as well as the actual teaching and grading of the various written assignments. Costs should also include all work that is required for intake of students, whether to process application forms or grade intake exams if such are used. Work that is required for graduation, including updating the credits registries, checking the completion of all required studies, and processing the certificates should also be included. Students are expected to gain work experience and professional supervision. The costs for these need to be considered. Student may already be employed in a suitable position for practicing their skills, or they may participate in organized practicum/internship periods. If they have supervisors or mentors that are not part of the teaching faculty, there may be a need to cover the costs.

A model where all costs fall upon the student is the least favorable from the student perspective. It can be problematic also from the society perspective because it leads to unfair disparity where only part of the psychologists have the financial opportunity to specialize. The selection may not lead into the best candidates getting trained. While health care providers need specialist expertise, employers in Finland have not been particularly active in covering program fees for their employees (Ministry of Education and Culture, 2019).

From the organizer perspective the financial model should provide stability, and funding from the governmental level (relevant ministries) offers this best. As funding from the Ministry of Education for master level tuition is generally secure for universities, developing a master program in clinical neuropsychology has been seen as a solution in e.g., France (Branco Lopes et al., 2021). This, however, is at odds with the EFPA EuroPsy model, where specialist training is defined at the post-master’s level (Lunt et al., 2015). Also for post-master training, Ministry of Education is the natural guiding ministry if the program is university-based. The necessary clinical training, on the other hand, takes place in health care which brings the program also within the sphere of the Ministry of Health. In Norway, the specialist training programs have developed fully under the guidance of health authorities organized by the Norwegian Psychological Association (Hessen et al., 2018a).

### Core Competencies and Subspecialties

The work field of clinical neuropsychology is wide ranging from diagnostic assessment and consultation to interventions and rehabilitation across all ages. The core competency requirements for all clinical neuropsychologist include in-depth knowledge and skills in: (a) general psychology including clinical psychology, ethical, and legal standards, (b) clinically relevant brain-behavioral relationships, (c) related clinical disciplines, (d) neuropsychological assessment, including decision-making and diagnostic competency according to current classification of diseases, (e) diversity and culture in relation to clinical neuropsychology, (f) communication of neuropsychological findings and results to relevant and diverse audiences, and (g) psychological and neuropsychological interventions, including treatment and rehabilitation (Rey-Casserly et al., 2012; Hessen et al., 2018b).

To appreciate the implications of childhood conditions to adulthood and old age it is important that the competencies cover comprehensive expert knowledge of clinical neuropsychology throughout the lifespan. Yet, clinicians usually work within a subspeciality of either pediatric or adult neuropsychology. This calls for a training model that provides possibilities to achieve the knowledge and skills required for the given subspeciality. In Finland, pediatric and adult neuropsychology are incorporated into the same curriculum, but professional expertise within a subspeciality can be ensured via personal study plan, where the students have the opportunity to select part of the courses based on their individual interests and emphasis.

Treatment and rehabilitation is an integral part of clinical neuropsychology along with assessment (Laaksonen, 1987; Norup et al., 2017; Turunen et al., 2019) and the need of professional neuropsychologists providing such services is growing (Kasten et al., 2021). Therefore, it is essential that the specialization training equally accounts for the foundational and functional competencies (e.g., theoretical basis, evidence-based methods) necessary for neuropsychological interventions. Given the limited number of clinical neuropsychologists practicing rehabilitation, it is also important to strengthen competencies regarding consultation for other health care professionals [see Ghag et al. (2021)] or in schools (Decker, 2008).

### Level of Research Emphasis

In the United States (US), the clinical neuropsychology training has been built upon the scientist-practitioner model which poses that professional psychologist could and should be both scientist and practitioner (Belar and Perry, 1992; Baker and Benjamin, 2000). The initial guidelines for training were explicated in the Houston Conference Policy Statement which defined the specialization to occur at doctoral, internship, and post-doctoral training levels (Hannay et al., 1998; Smith, 2019). In the current US framework, scientific knowledge and research skills are incorporated in the competencies that are required of a clinical neuropsychologist entering the profession (Rey-Casserly et al., 2012; Smith, 2019).

The doctorate requirement for specialists in clinical neuropsychology appears in a minority of countries, namely US, Canada, United Kingdom (UK), Ireland, and parts of Australia (Grote and Novitski, 2016; Hessen et al., 2018b; Hokkanen et al., 2019). In Finland, a doctorate is not a requirement for practicing in health care. In Scandinavia, of practitioners working in clinical neuropsychology, 11% were reported to have completed a Ph.D. and for Finland the figure was 14% (Norup et al., 2017).

Regardless of the associated degree requirements, the need for scientific bases of clinical action stems from the model of Evidence Based Practice (EBP) which, as applied in psychology and clinical neuropsychology, calls for empirically supported principles of psychological assessment, case formulation, therapeutic relationship, and intervention (APA Presidential Task Force on Evidence-Based Practice, 2006; Chelune, 2008). In the US competency framework of clinical neuropsychology, EBP is one of the key elements (Rey-Casserly et al., 2012; Smith, 2019). In comparing the competencies across several well-developed training programs, Hessen et al. (2018b) found the elements of EBP being well-represented in all of the seven countries reviewed.

If the doctorate track and the specialist track are separate, transfer from one to the other should be made as effortless as possible. If empirical research is a required competence in the specialist education taking a step further toward a doctorate is relatively small. And if the specialist program credits count also toward the doctoral degree, the step is made easier still. In Finland, the doctorate usually requires a minimum of three published research papers (plus a summary based on them published as a separate doctoral thesis) and 40 ECTS of study, and some of these requirements can be shared between the doctoral and the specialist track programs (Figure 1). A strategy selected by US, UK, Ireland, and Australia is to introduce a clinical doctorate, a Doctor of Psychology (PsyD), as an alternative. In Finland, the only available doctorate is a Ph.D. which is a research doctorate of 4 years full-time, following both the bachelor (3 years) and the master’s (2.5 years) degree. In the UK, the process involves a bachelor’s level education in psychology (3–4 years), and a Doctorate in Clinical Psychology (3 years) combining clinical practice, education, and research (Hessen et al., 2018b).

## Conclusion

An established specialist training model strengthens the position of the profession in health care. Planning the specialist training for clinical neuropsychology never happens in a vacuum, however. The existing health care system, legislation related to health care and education, competencies provided by the bachelor and master level studies in psychology, and the strategies of the stakeholder ministries must be taken into account. As experienced in Finland, these building blocks can also change over time. This paper provides information on different training models within clinical neuropsychology in Finland in the past 40 years. The experiences can be useful for other countries that are developing their models. The estimated need of practitioners and the educational resources including the available financial models for training differ between countries. The guiding principles in specialist training should focus on the advanced competencies expected from the neuropsychologist when entering the profession.

## Author Contributions

LH wrote the first draft of the manuscript and finalized it for submission. HJ, KR, TN, and EP had intellectual contributions to the content. All authors agreed to the submitted version of the publication.

## Conflict of Interest

LH was the Professor responsible for the clinical neuropsychology specialist program at the University of Helsinki, Finland. HJ and KR had 50% positions as Senior Lecturers in the same program. The remaining authors declare that the research was conducted in the absence of any commercial or financial relationships that could be construed as a potential conflict of interest.

## Publisher’s Note

All claims expressed in this article are solely those of the authors and do not necessarily represent those of their affiliated organizations, or those of the publisher, the editors and the reviewers. Any product that may be evaluated in this article, or claim that may be made by its manufacturer, is not guaranteed or endorsed by the publisher.

## Footnotes

1
http://www.ehea.info/page-three-cycle-system

2
www.europsy-efpa.eu

3
http://www.psykonet.fi/english

4
https://www.europsy.eu/quality-and-standards/europsy-specialisation
